# Beyond [^177^Lu]Lu-PSMA: a meta-analysis of safety and efficacy of emerging PSMA radioligand therapy agents

**DOI:** 10.1186/s12885-026-15900-y

**Published:** 2026-03-23

**Authors:** Ahmed Saad Abdlkadir, Dhuha Al-Adhami, Serin Moghrabi, Mike Machaba Sathekge, Michael Kreissl, Enrique Estrada-Lobato, Hongcheng Shi, Akram Al-Ibraheem

**Affiliations:** 1 Department of Nuclear Medicine, Baghdad Radiotherapy and Nuclear Medicine Hospital, Bab Al-Muadham, Baghdad, 10047 Iraq; 2 Department of Nuclear Medicine, King Hussein Cancer Center (KHCC), Queen Rania Street, Al Jubeiha, Amman, 11941 Jordan; 3 Department of Nuclear Medicine, University of Pretoria and Steve Biko Academic Hospital, Pretoria, 0001 South Africa; 4 Nuclear Medicine Research Infrastructure (NuMeRI), Steve Biko Academic Hospital, Pretoria, 0002 South Africa; 5 Department of Nuclear Medicine, Steve Biko Academic Hospital, Pretoria, 0001 South Africa; 6 Department of Radiology and Nuclear Medicine, Division of Nuclear Medicine, University Hospital of Magdeburg, Magdeburg, 39120 Germany; 7 Department of Nuclear Sciences and Applications, Nuclear Medicine and Diagnostic Imaging Section, Division of Human Health, International Atomic Energy Agency, Vienna, Austria; 8 Department of Nuclear Medicine, Zhongshan Hospital, Fudan University, Shanghai, China; 9 School of Medicine, University of Jordan, Al-Jubeiha, Amman, 11942 Jordan

**Keywords:** PSMA RLT, Prostate cancer, [²²⁵Ac]Ac-PSMA, [¹⁶¹Tb]Tb-PSMA, [¹³¹I]PSMA, Tandem therapy

## Abstract

**Background:**

This systematic review and meta-analysis evaluates the safety and efficacy of emerging prostate-specific membrane antigen (PSMA) radioligand therapy (RLT) agents used beyond [¹⁷⁷Lu]Lu-PSMA in metastatic castration-resistant prostate cancer (mCRPC).

**Methods:**

Systematic searches of PubMed, Web of Science, and Scopus were performed from inception until November 3, 2025. Studies reporting objective response rate (ORR), disease control rate (DCR), and/or toxicity outcomes were included. Meta-analytic pooling, assessment of publication bias, heterogeneity analyses, and subgroup evaluations were conducted using Stata software.

**Results:**

A total of 33 studies published between 2017 and 2025 met inclusion criteria, encompassing 3625 therapy cycles administered to 1525 patients. The pooled DCR was 86% (95% CI: 82–90%), and the pooled ORR was 57% (95% CI: 50–63%). [²²⁵Ac]Ac-PSMA monotherapy, evaluated in 17 studies, achieved pooled DCR and ORR values of 88% and 62%. Eight studies assessing [¹⁷⁷Lu]Lu/[²²⁵Ac]Ac-PSMA tandem therapy reported pooled DCR and ORR values of 84% and 51%. Five studies on [¹⁶¹Tb]Tb-PSMA demonstrated pooled DCR and ORR values of 81% and 46%. [¹³¹I]PSMA therapy, reported in three studies, resulted in a pooled DCR of 75% and pooled ORR of 48%. Adverse events were documented in 32 studies, with a pooled incidence of 26%. Most events were low-grade and reversible. Xerostomia and anemia were the most frequently reported toxicities, with xerostomia particularly associated with [²²⁵Ac]Ac-PSMA–containing regimens.

**Conclusion:**

These findings underscore the promising therapeutic potential of emerging PSMA RLT agents beyond [¹⁷⁷Lu]Lu-PSMA, with favorable biochemical responses and manageable safety profiles. Future large-scale prospective studies are essential to define optimal therapeutic roles and expand treatment opportunities for patients with mCRPC.

**Supplementary Information:**

The online version contains supplementary material available at 10.1186/s12885-026-15900-y.

## Introduction

Prostate cancer remains one of the most prevalent malignancies worldwide, ranking fourth in overall cancer incidence and second among men, with more than 1.4 million new cases reported in 2022 [1]. Although global incidence continues to rise, mortality has stabilized or declined in many high-income regions, reflecting improvements in early detection and systemic therapy [2]. Evidence from real-world studies indicates that approximately 20–30% of patients will progress to metastatic castration-resistant prostate cancer (mCRPC), typically within several years of developing hormone-sensitive state [3–5].

Significant advances in targeted radionuclide therapy (TRT) have led to the approval of prostate-specific membrane antigen (PSMA) radioligand therapy (RLT) for mCRPC [6], supported by compelling results from landmark clinical trials [7, 8]. [^177^Lu]Lu-PSMA RLT, administered in a six-cycle regimen at six-week intervals (6 × 6 protocol) [8], has demonstrated substantial clinical efficacy and a favorable safety profile, offering a new hope for patients who have exhausted conventional systemic therapies [9]. Emerging literature also suggests potential benefit from treatment continuation or rechallenge beyond the standard 6 × 6 regimen [10–12]. However, up to 30% of patients eventually develop resistance to [^177^Lu]Lu-PSMA, necessitating exploration of alternative therapeutic strategies [13–15].

To address this, several TRT agents are under investigation for patients who remain eligible for PSMA-targeted therapy. These include dual beta/Auger emitters such as [^161^Tb]Tb-PSMA, iodine-based agents such as [^131^I]PSMA, and alpha-emitting therapies such as [^225^Ac]Ac-PSMA, administered either as monotherapy or in combination with [^177^Lu]Lu-PSMA [16]. These agents possess distinct physical and radiobiological properties that may overcome resistance mechanisms and expand treatment options (Table 1). Despite growing interest, no comprehensive systematic review has synthesized their safety and efficacy. Therefore, this systematic review aims to evaluate these emerging PSMA-directed therapeutic approaches through pooled and subgroup meta-analyses.

**Table 1:** Physical and radiobiological properties of currently employed PSMA RLT in clinical practice

TRT agent	T_1/2_	☢	β Energy Range (keV)	σ Abundance	α Energy Range (MeV)	Advantage	Limitations
[^177^Lu]Lu-PSMA	6.7 d	β, γ, σ	60–500	Moderate	–	• Post-therapeutic imaging is feasible • The only approved and most widely used TRT for mCRPC.	• Possible resistance in some mCRPC patients after multiple cycles.
[^161^Tb]Tb-PSMA	6.9 d	β, γ, σ	50–600	High	–	• Post-therapeutic imaging is feasible • Abundant Auger electrons may target micrometastases.	• Evidence currently limited to early/observational studies.
[^131^I]PSMA	8 d	β, γ, σ	100–606	Low	–	• Post-therapeutic imaging is feasible • Long tissue penetration range can help eradicate bulky or heterogeneous disease.	• Mainly investigational • Thyroid blockade often needed • In vivo de-iodination may shorten residence time.
[^225^Ac]Ac-PSMA	10 d	α	–	–	5.8–8.4	• Very high LET and short-range cause potent DNA double-strand breaks.	• High xerostomia rates and other toxicities • Post-therapy imaging is challenging.
[^177^Lu]Lu/ [^225^Ac]Ac-PSMA	Up to 10 d	α, β, γ, σ	60–500	Moderate	5.8–8.4	• Post-therapeutic imaging feasible • Documented synergistic α/β effect.	• Evidence still limited to small observational series.

☢ Emission types, *α *Alpha particles, *σ *Auger electrons, *β *Beta particles, *γ *Gamma photons, *keV *Kiloelectron volt, *LET *Linear energy transfer, *mCRPC *Metastatic castration-resistant prostate cancer, *MeV *Megaelectron volt, *PSMA *Prostate-specific membrane antigen, *RLT *Radioligand therapy, *T1/2 *Physical half-life, *TRT *Targeted radionuclide therapy

## Materials and methods

This review was registered in the International prospective register of systematic reviews (PROSPERO ID: CRD420251242702). The meta-analysis was conducted in accordance with the most recent 2020 version of the Preferred Reporting Items for Systematic Reviews and Meta-Analyses (PRISMA) guidelines (Supplementary Table 1).

### Data sources and search strategy

Systematic literature searches of PubMed, Web of Science, and Scopus were performed from inception to November 3, 2025, to identify studies assessing the safety and efficacy of PSMA RLT beyond [^177^Lu]Lu-PSMA monotherapy in mCRPC. Three authors (ASA, DAA, and AA-I) independently executed the searches using MeSH and Emtree-based strategies (Supplementary Table 2). Inclusion criteria were restricted to original clinical studies evaluating PSMA RLT beyond [^177^Lu]Lu-PSMA monotherapy in patients with mCRPC. Studies were excluded if they were duplicates, non-original reports, book chapters, editorials, conference materials, preclinical investigations, dosimetry studies, or irrelevant to the review scope. All records were managed and organized using Microsoft Excel Professional Plus 2021 (Microsoft Corp., Redmond, Washington, US).

### Data collection

All studies satisfying the predefined inclusion criteria were systematically retrieved and analyzed. A dedicated Microsoft Excel spreadsheet was developed to ensure standardized data organization and review. Extracted variables included study authorship, publication year, country, article and study design, sample size, median age, previous therapies, PSMA RLT agent used, number of treatment cycles, treatment strategy and dose, biochemical indices, PSA-based biochemical response, and treatment-related toxicities graded using the Common Terminology Criteria for Adverse Events.

### Methodological quality

The quality of the included studies was thoroughly evaluated using the National Institutes of Health (NIH) Quality Assessment Tool for Observational Cohort and Cross-Sectional Studies [17]. This comprehensive assessment, consisting of 14 items, was independently applied by three authors (ASA, DAA, and AA-I) to determine each study’s methodological rigor and potential limitations. Based on the resulting numerical scores, studies were classified into three quality levels: Good (9–14 points), Fair (5–8 points), or Poor (0–4 points).

### Statistical analysis

Meta-analysis of pooled proportions—including the disease control rate (DCR), objective response rate (ORR), and adverse event rate—were summarized as point estimates with 95% confidence intervals (95% CI). The safety and efficacy of PSMA RLT agents, both collectively and individually, were evaluated and presented in tables and/or forest plots, provided that a minimum of two studies were available for the respective domain. Fixed-effects models were applied for outcomes supported by fewer than five studies, whereas random-effects models were used when five or more studies were available (Supplementary Table 3) [18]. Publication bias was evaluated using Egger’s test for domains with at least three contributing studies. For analyses based on random-effects modeling, heterogeneity was assessed using the I² statistic, with < 50% interpreted as low to moderate variation and > 50% as substantial to considerable variation [19]. Subgroup analyses were conducted when heterogeneity was substantial and at least ten studies were available for that domain [20]. The presence of heterogeneity between subgroups was evaluated using the Q test, and the corresponding p-value was used to determine statistical significance. A *p*-value < 0.05 was considered statistically significant. All statistical analyses were performed using Stata, version 17.0 (Stata Corporation, College Station, Texas, US).

## Results

**Fig. 1:**
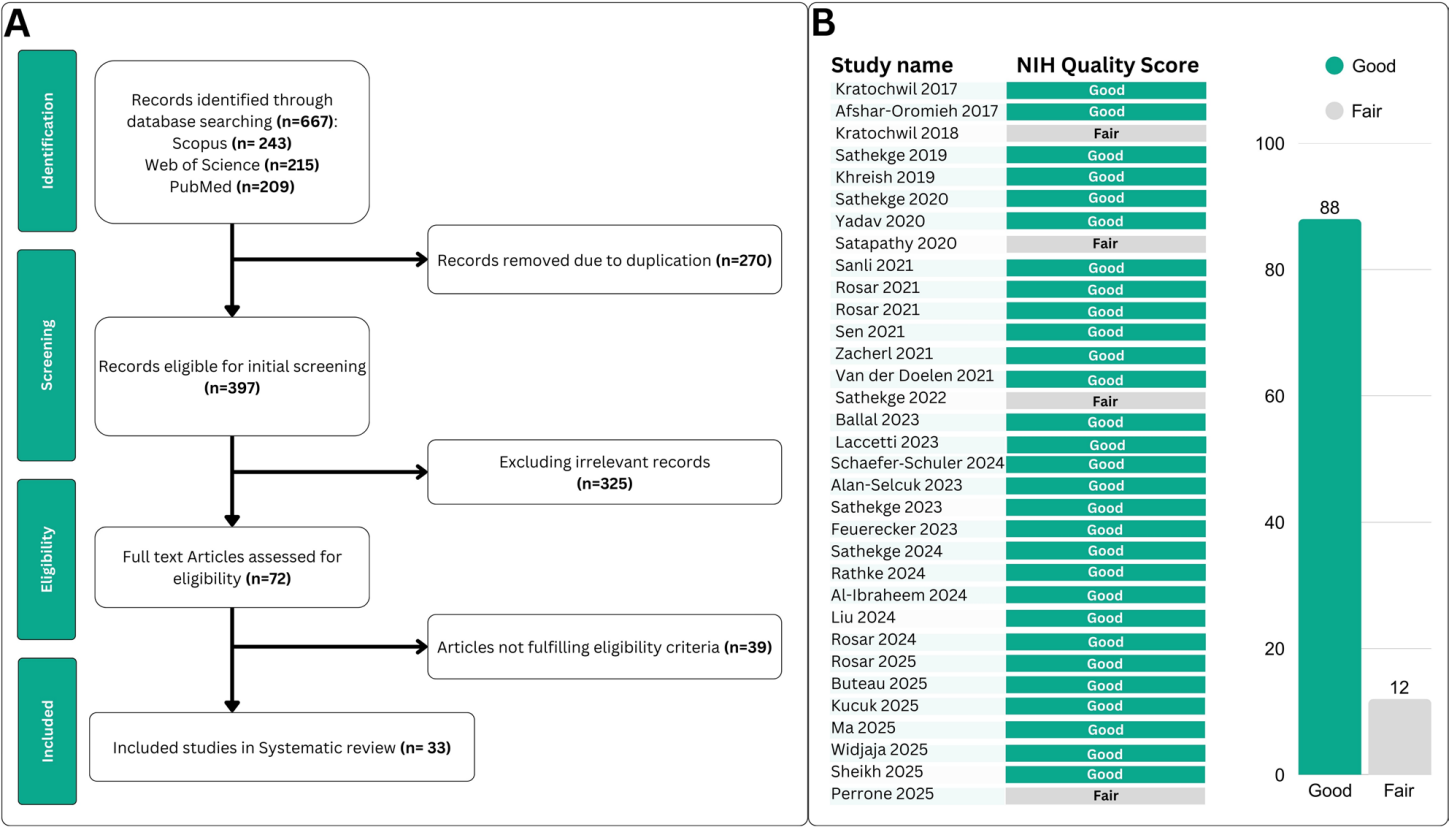
**A** Graphical flowchart illustrating study selection process. **B** Results of methodological quality evaluation, utilizing the NIH criteria

This systematic review initially identified 667 records across three databases. After removing 270 duplicates, 397 titles and abstracts were screened, with the majority excluded for not meeting the predefined objectives. Ultimately, 33 studies satisfied all eligibility criteria and were included in the systematic review and meta-analysis (Fig. 1A) [21–53].

### Methodological quality

The methodological quality of the included studies was evaluated using the NIH Quality Assessment Tool. Of the 33 studies incorporated into this systematic review and meta-analysis, 29 were rated as “good” [21, 22, 24–30, 32–34, 36–49, 51–53], and 4 were assessed as “fair” (Fig. 1B) [23, 31, 35, 50]. Detailed scoring for each study is provided in Supplementary Table 4.

### Systematic review

This systematic review included 33 studies published between 2017 and 2025, comprising 3625 PSMA RLT cycles administered to 1525 mCRPC patients [21–53]. The median age across study cohorts ranged from 62 to 77 years. Twenty-five studies used a retrospective design [21–26, 28–38, 40, 41, 43, 45, 49, 50, 52, 53], while eight adopted a prospective approach [27, 39, 42, 44, 46–48, 51]. PSMA-617 was the most frequently used radioligand, applied in 28 studies [22–33, 35–38, 40, 41, 43–46, 48–53]. Other formulations included PSMA-1095 in three studies [21, 39, 42], and PSMA-I&T in two studies [34, 47]. Most investigations employed cyclic dosing regimens [21–42, 44–53], with only one study using a fractionated dosing strategy [43]. Monotherapy was reported in 26 studies [21–24, 26, 27, 30–43, 45–49, 51], whereas eight studies implemented tandem therapy [25, 28, 29, 43, 44, 50, 52, 53]. Notably, one study evaluated both [^225^Ac]Ac-PSMA monotherapy (104 patients) and [^177^Lu]Lu/[^225^Ac]Ac-PSMA tandem therapy (129 patients) within the same cohort [43]. Geographically, nineteen studies were conducted in Europe [21–23, 25, 28–30, 33, 34, 36, 38, 43, 44, 46, 48, 50–53], five in South Africa [24, 26, 35, 40, 45], four in India [27, 31, 32, 37], two in North America [39, 42], one in China [49], one in Australia, and one in Jordan [41]. The key characteristics of the included studies are summarized in Table 2 and Supplementary Table 5.

**Table 2:** Characteristics of included studies

Study Name	Country	Patients	Age	Study Nature	TRT agent
Kratochwil 2017 [22]	DE	14	68	OR	[^225^Ac]Ac-PSMA-617
Afshar-Oromieh 2017 [21]	DE	34	75	OR	[^131^I]PSMA-1095
Kratochwil 2018 [23]	DE	40	70	OR	[^225^Ac]Ac-PSMA-617
Sathekge 2019 [24]	ZA	17	65	OR	[^225^Ac]Ac-PSMA-617
Khreish 2020 [25]	DE	20	72	OR	[^177^Lu]/[^225^Ac]Ac-PSMA-617
Sathekge 2020 [26]	ZA	73	69	OR	[^225^Ac]Ac-PSMA-617
Yadav 2020 [27]	IN	28	70	OP	[^225^Ac]Ac-PSMA-617
Satapathy 2021 [31]	IN	11	68	OR	[^225^Ac]Ac-PSMA-617
Sanli 2021 [30]	TR	12	70	OR	[^225^Ac]Ac-PSMA-617
Rosar 2021 [28]	DE	17	69	OR	[^177^Lu]Lu/[^225^Ac]Ac-PSMA-617
Rosar 2021 [29]	DE	15	77	OR	[^177^Lu]Lu/[^225^Ac]Ac-PSMA-617
Sen 2021 [32]	IN	38	68	OR	[^225^Ac]Ac-PSMA-617
Zacherl 2021 [34]	DE	14	75	OR	[[^225^Ac]Ac-PSMA-I&T
Van der Doelen 2021	NL	13	71	OR	[^225^Ac]Ac-PSMA-617
Sathekge 2022 [35]	ZA	53	63	OR	[^225^Ac]Ac-PSMA-617
Laccetti 2023 [39]	US	9	70	OP	[^131^I]PSMA-1095
Ballal 2023 [37]	IN	56	67	OR	[^225^Ac]Ac-PSMA-617
Alan-Selcuk 2023 [36]	TR	23	70	OR	[^225^Ac]Ac-PSMA-617
Schaefer-Schuler 2024 [46]	DE	6	74	OP	[^161^Tb]Tb-PSMA-617
Sathekge 2023 [40]	ZA	21	67	OR	[^225^Ac]Ac-PSMA-617
Feuerecker 2023 [38]	DE	21	69	OR	[^225^Ac]Ac-PSMA-617
Sathekge 2024 [45]	ZA	488	68	OR	[^225^Ac]Ac-PSMA-617
Rathke 2024 [43]	CH	233	62	OR	[^225^Ac]Ac-PSMA-617
Liu 2024 [42]	CA	11	74	OP	[^131^I]PSMA-1095

♠ [^225^Ac]Ac-PSMA monotherapy was utilized in 104 patients and [^177^Lu]Lu/[^225^Ac]Ac-PSMA tandem therapy was implemented in 129 patients within the same cohort, *AU *Australia, *CA *Canada, *CH *Switzerland, *CN *China, *DE *Germany, *IN *India, *JO *Jordan, *NL *Netherlands, *OP *Original prospective, *OR *Original retrospective, *PSMA *Prostate-specific membrane antigen, *TR *Turkey, *TRT *Targeted radionuclide therapy, *US *United States, *ZA *South Africa

### Meta-analysis

#### Biochemical efficacy of PSMA RLT agents beyond [177Lu]Lu-PSMA

**Fig. 2:**
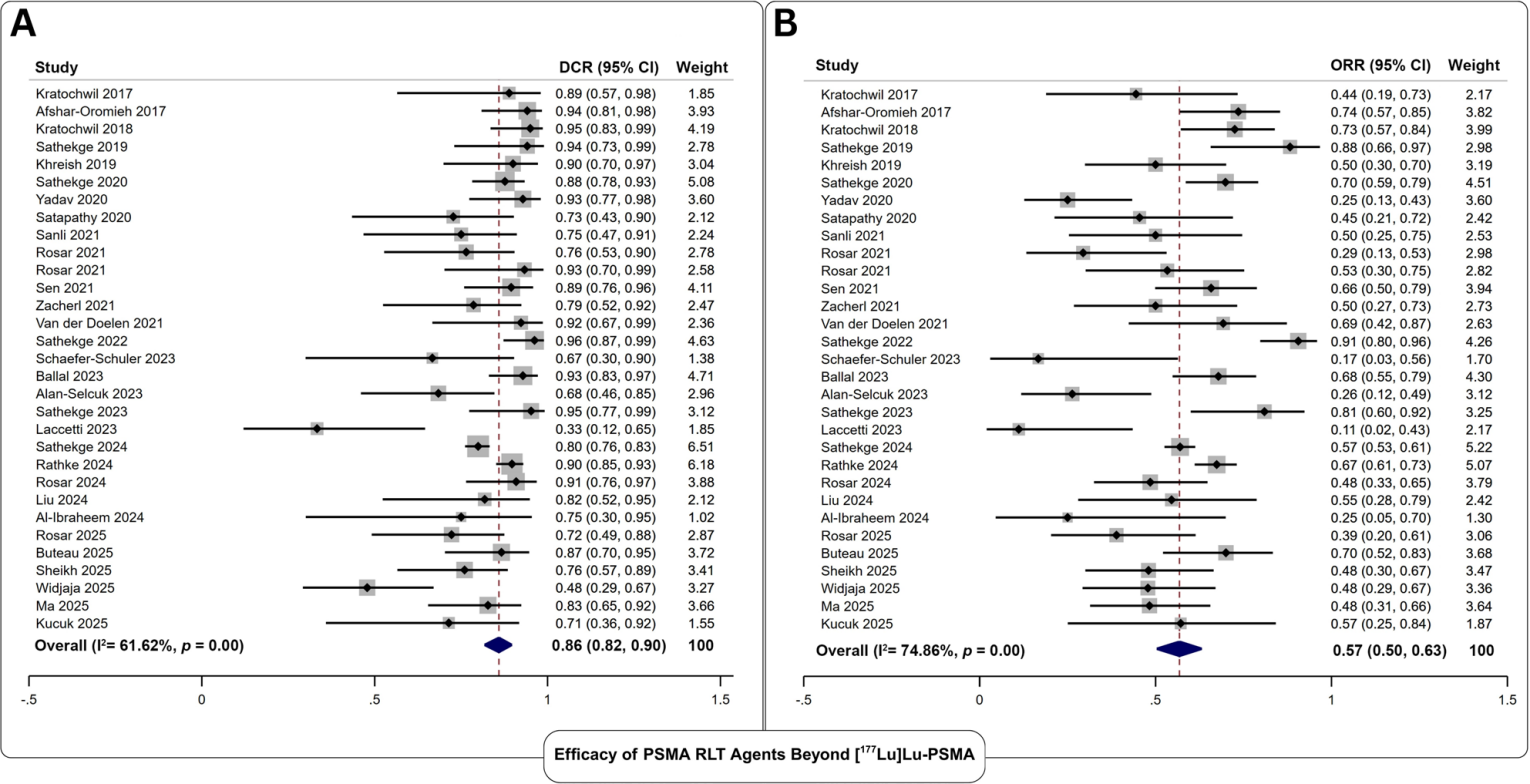
Forest plot analysis of **A** disease control rate, and **B** objective response rate for the assessment of biochemical efficacy of PSMA RLT Agents Beyond [^177^Lu]Lu-PSMA

Thirty-one studies reported biochemical responses following 3453 PSMA RLT cycles administered to 1406 patients with mCRPC (Fig. 2). The pooled DCR was 86% (95% CI: 82–90%), and the pooled ORR was 57% (95% CI: 50–63%). No significant publication bias was detected (*p*>0.11 for both outcomes). Substantial heterogeneity was observed, with I² values exceeding 60% for both endpoints. Differences in sample size and the use of multiple therapeutic radiopharmaceuticals contributed significantly to between-study heterogeneity for DCR and ORR (Q>3.2; p≤0.05). Retrospective studies demonstrated a markedly higher ORR than prospective studies, emerging as a key source of subgroup heterogeneity (61% vs. 41%; *p*=0.02). Subgroup meta-analysis results are summarized in Table 3.

**Table 3:** Results of subgroup meta-analysis for all applied PSMA RLT agents beyond [177Lu]Lu-PSMA

Covariates	Category/Estimate	Studies	DCR% (95% CI)	Q Test (*p*)	ORR% (95% CI)	Q Test (*p*)
Study Design	Retrospective	23	87 (83-91)	1.2 (0.27)	61 (55-68)	5.6 (0.02)
	Prospective	8	81 (67-92)		41 (27-57)	
Age Group	Geriatrics	17	82 (73-89)	2.5 (0.09)	57 (50-63)	3.1 (0.06)
	Middle Age	14	90 (85-94)		66 (57-73)	
Sample Size	< 30 participants	21	80 (73-87)	8.7 (0.01)	47 (37-56)	4.2 (0.03)
	≥ 30 participants	10	90 (86-94)		69 (61-75)	
Baseline PSA	≥ 100 ng/ml	25	87 (82-91)	1.1 (0.31)	56 (50-63)	0.1 (0.95)
	< 100 ng/ml	6	81 (66-93)		56 (35-76)	
Heavy Pretreatment	≥ 3 Therapy lines	26	83 (82-90)	0.1 (0.78)	56 (49-63)	0.31 (0.58)
	< 3 Therapy lines	5	86 (82-90)		60 (41-80)	
TRT emission	α	17	88 (84-92)	3.3 (0.05)	62 (54-70)	3.9 (0.04)
	α/β	7	84 (70-94)		51 (41-63)	
	β/Auger	8	75 (60-91)		46 (26-66)	

#### Safety of PSMA RLT agents beyond [^177^Lu]Lu-PSMA

Adverse events were reported in 32 studies after 3388 PSMA RLT cycles administered to 1390 mCRPC patients (Table 4). The pooled adverse event rate was 26% (95% CI: 22–31%). Low-grade xerostomia and fatigue were the most common clinical toxicities, with a combined frequency exceeding 54%. Low-grade anemia was the most frequently reported biochemical toxicity (pooled rate: 41%). High-grade xerostomia was the most prevalent grade ≥ 3 toxicity (11%). Additional, less common adverse events are listed in Supplementary Table 6.

**Table 4:** Common Clinical and biochemical adverse events reported following PSMA RLT administration

TRT Agent	Low grade toxicity, Estimate (95% CI)			High grade toxicity, Estimate (95% CI)		
	Most common	2nd most	3rd most	Most common	2nd most	3rd most
**Clinically prevalent adverse events**						
All PSMA RLT	Xerostomia G1–2: 68%	Fatigue G1–2: 55%	GIT G1–2: 31%	Xerostomia G3: 11%	Bone pain flare: 10%	Fatigue G3–4: 3%
[^225^Ac]Ac-PSMA	Xerostomia G1–2: 82%	Fatigue G1–2: 68%	GIT G1–2: 44%	Xerostomia G3: 12%	–	–
[^177^Lu]Lu/[^225^Ac]Ac-PSMA	Xerostomia G1–2: 42%	–	–	–	–	–
[^161^Tb]Tb-PSMA	Fatigue G1–2: 55%	Xerostomia G1–2: 54%	GIT G1–2: 10%	Bone pain flare: 10%	–	–
[^131^I]PSMA	Xerostomia G1–2: 51%	GIT G1–2: 24%	Fatigue G1–2: 13%	–	–	–
**Biochemically prevalent adverse events**						
All PSMA RLT	Anemia G1–2: 41%	Leukopenia G1–2: 21%	Thrombocytopenia G1–2: 21%	Anemia G3–4: 10%	Thrombocytopenia G3–4: 7%	Nephrotoxicity G3–4: 4%
[^225^Ac]Ac-PSMA	Anemia G1–2: 40%	Nephrotoxicity G1–2: 24%	Leukopenia G1–2: 18%	Anemia G3–4: 9%	Thrombocytopenia G3–4: 5%	Nephrotoxicity G3–4: 3%
[^177^Lu]Lu/[^225^Ac]Ac-PSMA	Anemia G1–2: 35%	Thrombocytopenia G1–2: 23%	Leukopenia G1–2: 22%	Anemia G3–4: 10%	Thrombocytopenia G3–4: 6%	Leukopenia G3–4: 3%
[^161^Tb]Tb-PSMA	Anemia G1–2: 56%	Leukopenia G1–2: 31%	Thrombocytopenia G1–2: 25%	Anemia G3–4: 16%	Thrombocytopenia G3–4: 11%	–
[^131^I]PSMA	Leukopenia G1–2: 32%	Thrombocytopenia G1–2: 29%	GIT G1–2: 24%	Thrombocytopenia G3–4: 19%	Leukopenia G3–4: 6%	–

*CI* Confidence interval, *G1–2 *Grade 1–2, *G3–4 *Grade 3–4, *GIT *Gastrointestinal toxicity, *PSMA *Prostate‑specific membrane antigen, *RLT *Radioligand therapy, *TRT *Targeted radionuclide therapy

### Subgroup meta-analysis of RLT agents

#### Biochemical efficacy of [^225^Ac]Ac-PSMA monotherapy

**Fig. 3:**
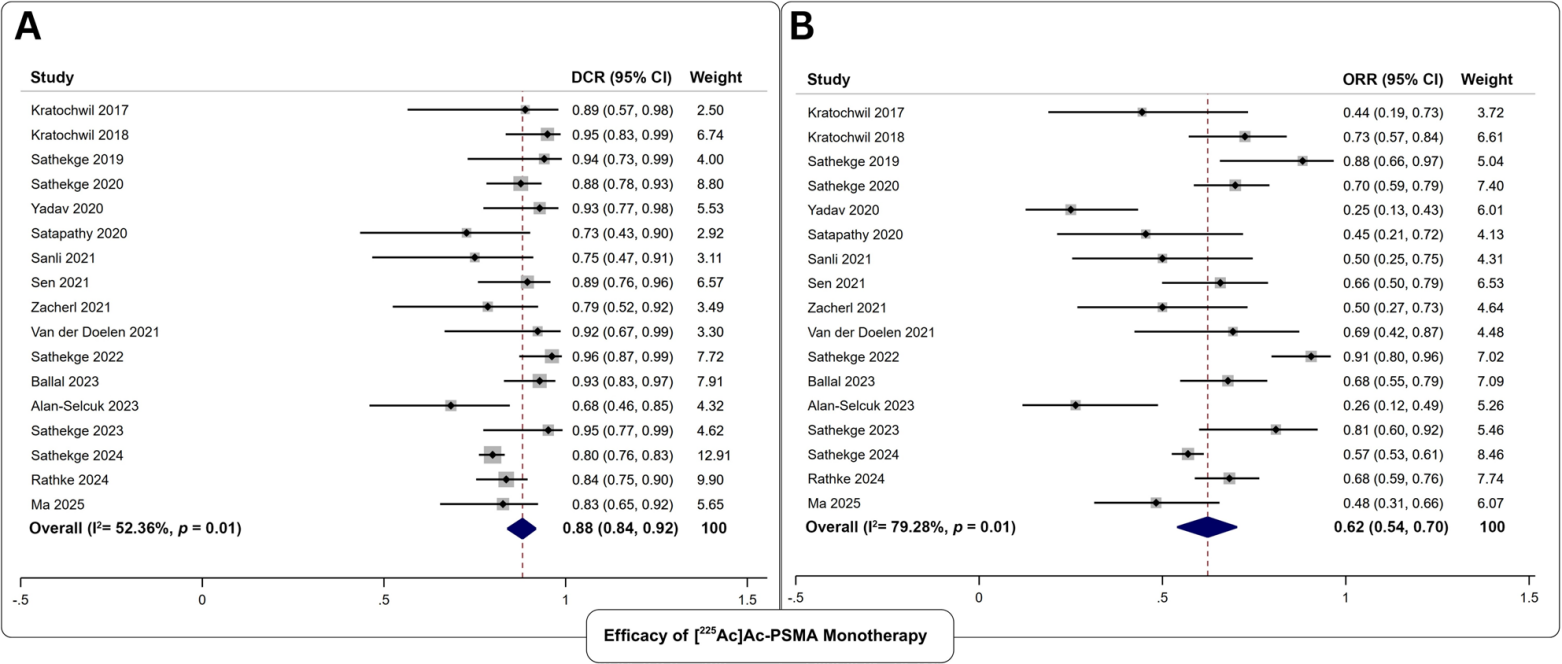
Forest plot analysis of **A** disease control rate, and **B** objective response rate for the assessment of biochemical efficacy of [^225^Ac]Ac-PSMA Monotherapy

Seventeen studies reported outcomes following 2645 [^225^Ac]Ac-PSMA monotherapy cycles administered to 1015 mCRPC patients (Fig. 3). The pooled DCR was 88% (95% CI: 84–92%), and the pooled ORR was 62% (95% CI: 54–70%). No significant publication bias was detected (*p*;>;0.39). Substantial heterogeneity was observed for both endpoints (I² >52%).

#### Safety of [^225^Ac]Ac-PSMA monotherapy

Across 17 studies involving 2626 treatment cycles in 1025 patients, the pooled adverse event rate was 28% (95% CI: 21–34%). Low-grade xerostomia and fatigue were the most frequent clinical toxicities, with pooled frequencies of 82% and 68%, respectively (Table 4). Low-grade anemia was the most common biochemical toxicity (41%). High-grade xerostomia was the predominant grade ≥ 3 event (11%). Additional adverse events are detailed in Supplementary Table 6.

#### Biochemical efficacy of [^177^Lu]Lu/[^225^Ac]Ac-PSMA tandem therapy

**Fig. 4:**
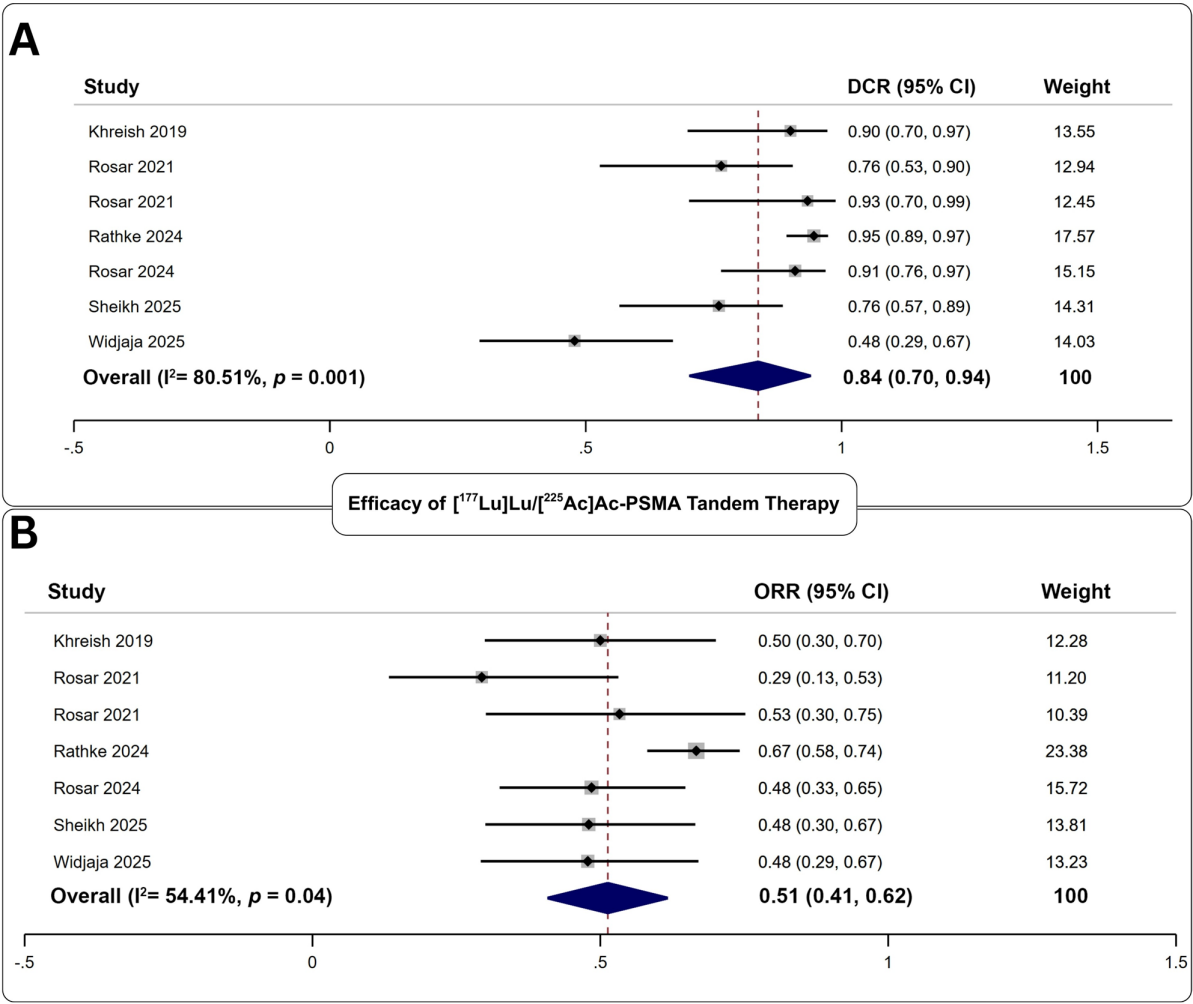
Forest plot analysis of **A** disease control rate, and **B** objective response rate for the assessment of biochemical efficacy of [^177^Lu]Lu/[^225^Ac]Ac-PSMA tandem therapy

Eight studies evaluated 681 tandem cycles in 262 patients (Fig. 4). The pooled DCR was 84% (95% CI: 70–94%), and the pooled ORR was 51% (95% CI: 41–62%). No significant publication bias was identified (*p*;>;0.41). Heterogeneity remained substantial (I² >54%).

#### Safety of [^177^Lu]Lu/[^225^Ac]Ac-PSMA tandem therapy

Seven studies reported toxicity outcomes following 616 treatment cycles in 326 patients. The pooled adverse event rate was 20% (95% CI: 10–33%). Low-grade xerostomia was the only consistently reported clinical toxicity (pooled rate: 42%). Low-grade anemia was the most common biochemical event (35%), while high-grade anemia was the predominant severe toxicity at 10% cumulative rate (Table 4). Additional adverse events are reported in Supplementary Table 6.

#### Biochemical Efficacy of [^161^Tb]Tb-PSMA

**Fig. 5:**
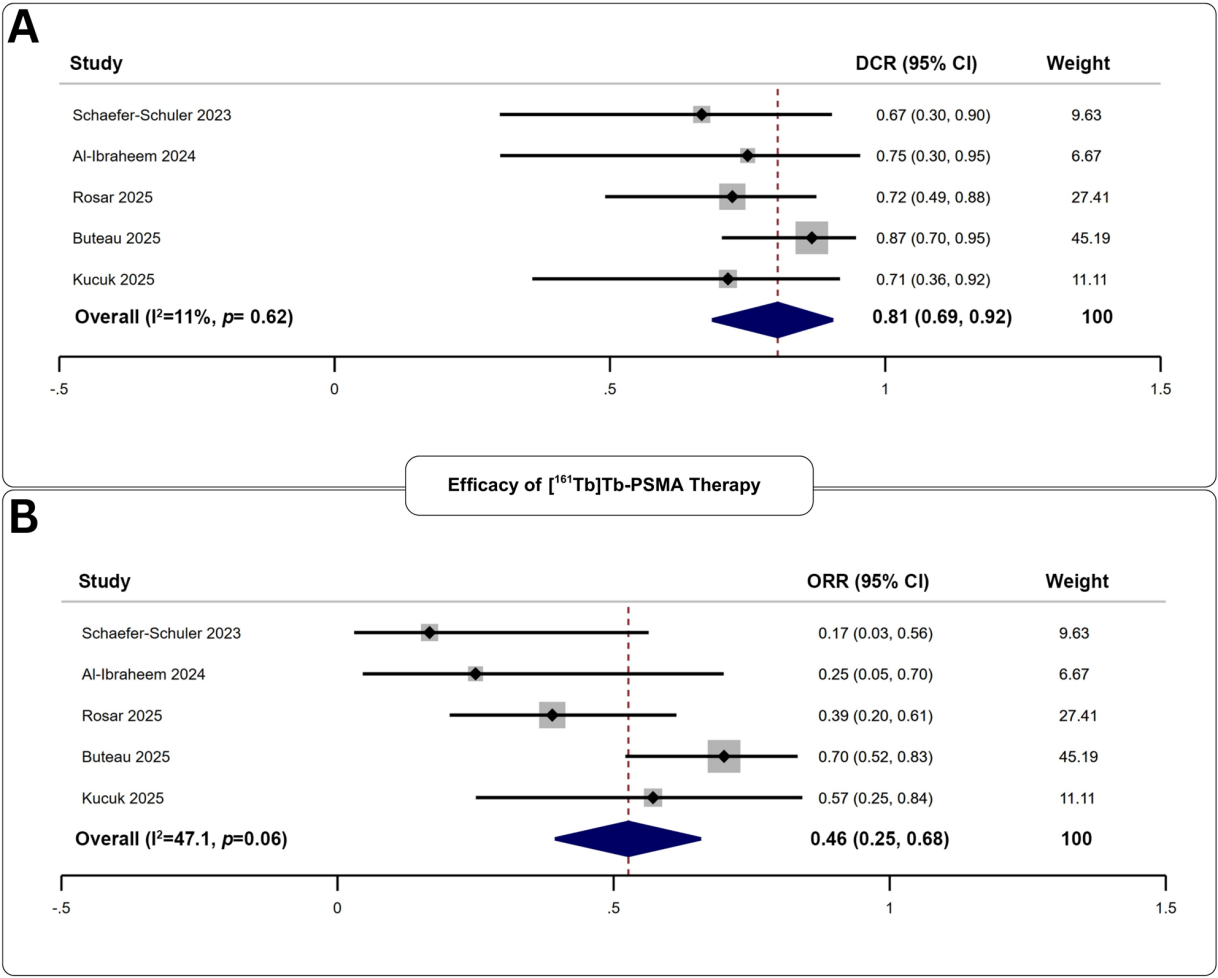
Forest plot analysis of **A** disease control rate, and **B** objective response rate for the assessment of biochemical efficacy of [^161^Tb]Tb-PSMA therapy

Five studies reported outcomes following 203 [^161^Tb]Tb-PSMA cycles administered to 65 patients (Fig. 5). The pooled DCR was 81% (95% CI: 69–92%), and the pooled ORR was 46% (95% CI: 25–68%). No significant heterogeneity or publication bias was detected (*p*;>;0.06 for both).

#### Safety of [^161^Tb]Tb-PSMA

Across five studies involving 203 cycles, the pooled adverse event rate was 24% (95% CI: 14–33%). Low-grade fatigue was the most common clinical toxicity (55%). Low-grade anemia was the most frequently reported biochemical event (56%). High-grade anemia (Table 4), was the predominant severe toxicity (16%). Additional events are summarized in Supplementary Table 6.

#### Biochemical Efficacy of [^131^I]PSMA

**Fig. 6:**
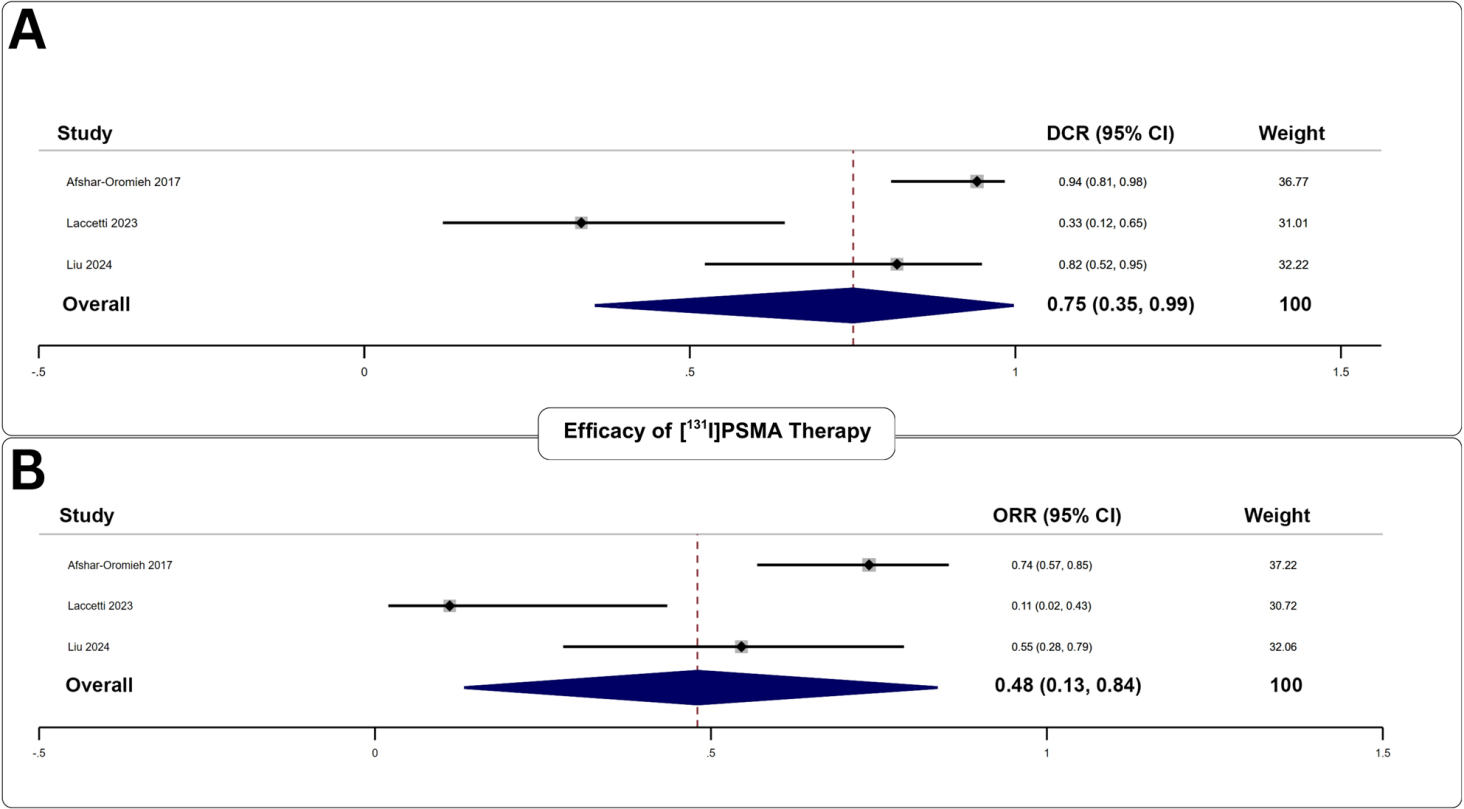
Forest plot analysis of **A** disease control rate, and **B** objective response rate for the assessment of biochemical efficacy of [^131^I]PSMA therapy

Three studies examined 95 [^131^I]PSMA cycles administered to 54 patients (Fig. 6). The pooled DCR was 75% (95% CI: 69–92%), and the pooled ORR was 48% (95% CI: 25–68%), with no significant publication bias (*p*;>;0.76).

#### Safety of [^131^I]PSMA

Adverse events were reported in three studies following 95 treatment cycles in 54 patients. The pooled adverse event rate was 25% (95% CI: 14–38%). Low-grade xerostomia was the most frequent clinical toxicity (51%). Low-grade leukopenia was the most common biochemical toxicity (32%). High-grade thrombocytopenia was the predominant severe toxicity at 19% (Table 4). Additional adverse events are presented in Supplementary Table 6.

## Discussion

Integrating the most recent evidence, our systematic review and meta-analysis demonstrated that a substantial proportion of patients achieve disease control benefits following PSMA RLT, with a cumulative DCR of 86% and an ORR of 57%. Despite a history of heavy pretreatment reported in the majority of studies [22–38, 41, 43–46, 48–53], a high safety profile is observed, characterized by a low rate of manageable toxicity, with pooled rates of up to 26% across all PSMA RLT agents.

[^225^Ac]Ac-PSMA monotherapy is the most extensively studied alternative agent beyond [^177^Lu]Lu-PSMA [22–24, 26, 27, 30–34, 36–38, 43–46, 49], and is frequently administered in cases of [^177^Lu]Lu-PSMA resistance or aggressive clinical scenarios such as fulminant bone marrow metastases [54, 55]. Seventeen out of eighteen available studies on [^225^Ac]Ac-PSMA monotherapy reported pooled DCRs and ORRs of 82% and 68%, confirming the promising therapeutic potential of α-particle therapy [22–24, 26, 27, 30–34, 36, 37, 43–46, 49]. However, xerostomia remains a major dose-limiting toxicity, with an overall pooled prevalence of 82% for low-grade xerostomia and 11% for grade 3 xerostomia [22–24, 26, 27, 30–34, 36–38, 43–46, 49]. According to a recent meta-analysis of [^225^Ac]Ac-PSMA-induced xerostomia, comparative evidence suggests that [^177^Lu]Lu/[^225^Ac]Ac-PSMA tandem therapy substantially reduces xerostomia risk, supporting its potential role in minimizing salivary toxicity [56].

The last few years have witnessed the implementation of this first combined dual RLT approach through the coadministration of [^177^Lu]Lu-PSMA with [^225^Ac]Ac-PSMA [25]. Under the tandem therapy concept, this approach is documented to achieve a synergistic effect by providing diverse energy ranges, dual potencies, and varied emission spectra [57]. Typically, implemented in a 50% [^177^Lu]Lu-PSMA/50% [^225^Ac]Ac-PSMA dosage formulation, it promises to provide the dual efficacy benefit of both agents while minimizing toxicity with a reduced dose for each agent. Our meta-analysis of the seven currently available studies revealed that a pooled DCR and ORR of 82% and 68%, respectively, were achieved [25, 28, 29, 43, 44, 50, 52, 53]. Importantly, a lower rate of xerostomia (42%), with no evident high-grade xerostomia, seems promising. This mandates further large-scale prospective studies to potentially substantiate the efficacy and safety profile of this unique therapeutic approach, especially when it is reserved for highly resistant refractory cases.

Following the recent first-in-human study of [^161^Tb]Tb-PSMA [58], several preliminary studies have been launched with the main aim of replicating the success of [^177^Lu]Lu-PSMA. These two PSMA RLT agents share great semblance, but [^161^Tb]Tb-PSMA offers the additional benefit of the potential to eradicate micrometastases through its higher Auger electron abundance, making it a more compelling substitute for [^177^Lu]Lu-PSMA, particularly for patients with micrometastatic disease [59]. Current evidence, on the basis of this meta-analysis, suggests that [^161^Tb]Tb-PSMA can indeed replicate the success story of [^177^Lu]Lu-PSMA, achieving an 81% DCR and 46% ORR [41, 46–48, 51]. Notably, high rates of low-grade anemia, fatigue, and xerostomia were found, exceeding 50%. However, most current studies utilized [^161^Tb]Tb-PSMA in a heavily pretreated mCRPC population, enrolling even those who had prior [^225^Ac]Ac-PSMA RLT. Consequently, some of these studies acknowledge that the toxicity rate might be confounded by prior multiple therapy lines, with xerostomia even being reported as stable or induced by [^225^Ac]Ac-PSMA rather than [^161^Tb]Tb-PSMA itself [46, 51]. Uniquely, this agent was also reported to be associated with a bone pain flare rate of 10% [46, 48]. Such striking findings need to be acknowledged, as this might complicate interim imaging analysis. Future studies should aim to prioritize treatment with [^161^Tb]Tb-PSMA as a frontline PSMA RLT agent to accurately appreciate the true rate of adverse events.

Owing to its high tissue penetration range, wide radionuclide availability, and ease of [^131^I] production, [^131^I]PSMA is another emerging PSMA RLT agent currently under investigation [16]. In a total of three studies conducted to evaluate its safety and efficacy, [^131^I]PSMA achieved a pooled DCR of 75% and a pooled ORR of 48% [21, 39, 42]. Associated toxicities included 51% low-grade xerostomia and 32% low-grade leukopenia. Thyroid blockade is surely needed to prevent unnecessary RLT-induced hypothyroidism [21, 39, 42]. Current evidence is limited by the small sample size and limited number of studies. Thus, further large-scale prospective trials are eagerly awaited to substantiate its evidence, especially since [^131^I]PSMA will serve to provide an alternative production route for PSMA RLT in the face of current supply/demand issues driven by the sole reliance on the limited production of [^177^Lu]Lu-PSMA [60, 61].

Among all achieved response patterns, the partial response rate was the most prevalent across all administered PSMA RLT agents, resulting in an ORR of approximately 50–60% for each applied PSMA RLT agent [21–37, 39–49, 51–53]. Although seldom reported, biochemical complete response (CR) was acknowledged in three studies in a minority of patients, indicating that non-[^177^Lu]Lu-PSMA agents can exert optimal biochemical responses [21, 35, 40], in line with what has been occasionally achieved in some patients following [^177^Lu]Lu-PSMA [62, 63].

While biochemical efficacy has been the main core of this meta-analysis, imaging-based response and the impact on quality of life represent other useful clinical metrics to provide insight into treatment efficacy. Unfortunately, it was not possible to evaluate all of these metrics due to unavailability and/or a lack of unified response criteria to make them eligible for meta-analyses. Future studies should focus on providing a uniform evaluation format for quality of life and imaging response, adhering to currently provided guiding principles from organizations such as the European Association of Urology (EAU), the Prostate Cancer Working Group (PCWG), the European Association of Nuclear Medicine (EANM), and the Society of Nuclear Medicine and Molecular Imaging (SNMMI), among others [64–66].

Another potential limitation to acknowledge is the abundance of retrospective studies and monocentric experience, alongside the presence of significant heterogeneity across most of the examined domains. This was typically addressed through subgroup meta‑analyses to identify potential confounders. Nevertheless, the very high heterogeneity observed in several pooled analyses warrants careful interpretation of the overall findings. Differences in treatment lines, prior PSMA RLT exposure, administered activity, and inconsistent response definitions may have influenced the pooled outcomes, limiting their generalizability. These variations highlight the need for standardized protocols and prospective multicenter comparisons to consolidate the evidence base. Despite these limitations, our meta-analysis is the first and only study to inform theranosticians and oncologists about the potential promise of PSMA RLT agents beyond [^177^Lu]Lu-PSMA.

## Conclusion

This systematic review and meta-analysis outlines the potential promise of emerging PSMA RLT agents. Beyond [^177^Lu]Lu-PSMA monotherapy, rivaling TRT agents leveraging same molecular targeting demonstrate remarkable antitumor efficacy and favorable safety profiles, underscoring their potential to diversify PSMA-targeted therapeutic options for the same indication. TRT agents with unique physical characteristics—such as alpha-emitting constructs (e.g., [^225^Ac]Ac-PSMA), tandem alpha/beta combinations (e.g., [^177^Lu]Lu/[^225^Ac]Ac-PSMA), and auger-rich isotopes (e.g., [^161^Tb]Tb-PSMA)—may broaden therapeutic applicability by exploiting complementary radiobiologic effects. Additionally, [^131^I]PSMA could offer a viable alternative in settings challenged by supply constraints of established radionuclides. Future studies should prioritize large-scale, multicenter clinical evaluations to validate these promising findings and further define their role within the evolving PSMA RLT landscape.

## Supplementary Information

Supplementary Material 1.

## Data Availability

The data that support the findings of this study are not openly available due to reasons of sensitivity and are available from the corresponding author upon reasonable request.

## Abbreviations

CR: Complete response
DCR: Disease control rate
EANM: European Association of Nuclear Medicine
EAU: European Association of Urology
mCRPC: Metastatic castration-resistant prostate cancer
NIH: National Institutes of Health
ORR: Objective response rate
PCWG: Prostate Cancer Working Group
PRISMA: Preferred Reporting Items for Systematic Reviews and Meta-Analyses
PROSPERO: International Database for the Prospective Registration of Systematic Reviews
PSMA: Prostate-specific membrane antigen
RLT: Radioligand therapy
SNMMI: Society of Nuclear Medicine and Molecular Imaging
TRT: Targeted radionuclide therapy
