# Supplementary material for: Beyond [177Lu]Lu-PSMA: a meta-analysis of safety and efficacy of emerging PSMA radioligand therapy agents
Source: BMC Cancer. 2026 Mar 23;26:551. doi: 10.1186/s12885-026-15900-y (PMC13134245; doi:10.1186/s12885-026-15900-y)
Supplement: Supplementary file 1 — Supplementary Material 1. [file 12885_2026_15900_MOESM1_ESM.pdf]

Supplementary Table 1: Filled PRSIMA Protol checkelist

Section and Topic	Item #	Checklist item	Page #
**TITLE**			1
Title	1	Identify the report as a systematic review.	✓
**ABSTRACT**			1
Abstract	2	See the PRISMA 2020 for Abstracts checklist.	✓
**INTRODUCTION**			5
Rationale	3	Describe the rationale for the review in the context of existing knowledge.	✓
Objectives	4	Provide an explicit statement of the objective(s) or question(s) the review addresses.	✓
**METHODS**			6-8
Eligibility criteria	5	Specify the inclusion and exclusion criteria for the review and how studies were grouped for the syntheses.	✓
Information sources	6	Specify all databases, registers, websites, organisations, reference lists and other sources searched or consulted to identify studies. Specify the date when each source was last searched or consulted.	✓
Search strategy	7	Present the full search strategies for all databases, registers and websites, including any filters and limits used.	✓
Selection process	8	Specify the methods used to decide whether a study met the inclusion criteria of the review, including how many reviewers screened each record and each report retrieved, whether they worked independently, and if applicable, details of automation tools used in the process.	✓
Data collection process	9	Specify the methods used to collect data from reports, including how many reviewers collected data from each report, whether they worked independently, any processes for obtaining or confirming data from study investigators, and if applicable, details of automation tools used in the process.	✓
Data items	10a	List and define all outcomes for which data were sought. Specify whether all results that were compatible with each outcome domain in each study were sought (e.g. for all measures, time points, analyses), and if not, the methods used to decide which results to collect.	✓
	10b	List and define all other variables for which data were sought (e.g. participant and intervention characteristics, funding sources). Describe any assumptions made about any missing or unclear information.	✓
Study risk of bias assessment	11	Specify the methods used to assess risk of bias in the included studies, including details of the tool(s) used, how many reviewers assessed each study and whether they worked independently, and if applicable, details of automation tools used in the process.	✓
Effect measures	12	Specify for each outcome the effect measure(s) (e.g. risk ratio, mean difference) used in the synthesis or presentation of results.	✓
Synthesis methods	13a	Describe the processes used to decide which studies were eligible for each synthesis (e.g. tabulating the study intervention characteristics and comparing against the planned groups for each synthesis (item #5)).	✓
	13b	Describe any methods required to prepare the data for presentation or synthesis, such as handling of missing summary statistics, or data conversions.	✓
	13c	Describe any methods used to tabulate or visually display results of individual studies and syntheses.	✓
	13d	Describe any methods used to synthesize results and provide a rationale for the choice(s). If meta-analysis was performed, describe the model(s), method(s) to identify the presence and extent of statistical heterogeneity, and software package(s) used.	✓
	13e	Describe any methods used to explore possible causes of heterogeneity among study results (e.g. subgroup analysis, meta-regression).	✓
	13f	Describe any sensitivity analyses conducted to assess robustness of the synthesized results.	✓
Reporting bias assessment	14	Describe any methods used to assess risk of bias due to missing results in a synthesis (arising from reporting biases).	✓
Certainty assessment	15	Describe any methods used to assess certainty (or confidence) in the body of evidence for an outcome.	✓
**RESULTS**			9-13
Study selection	16a	Describe the results of the search and selection process, from the number of records identified in the search to the number of studies included in the review, ideally using a flow diagram.	✓
	16b	Cite studies that might appear to meet the inclusion criteria, but which were excluded, and explain why they were excluded.	✓
Study characteristics	17	Cite each included study and present its characteristics.	✓
Risk of bias in studies	18	Present assessments of risk of bias for each included study.	✓
Results of individual studies	19	For all outcomes, present, for each study: (a) summary statistics for each group (where appropriate) and (b) an effect estimate and its precision (e.g. confidence/credible interval), ideally using structured tables or plots.	✓
Results of syntheses	20a	For each synthesis, briefly summarise the characteristics and risk of bias among contributing studies.	✓
	20b	Present results of all statistical syntheses conducted. If meta-analysis was done, present for each the summary estimate and its precision (e.g. confidence/credible interval) and measures of statistical heterogeneity. If comparing groups, describe the direction of the effect.	✓
	20c	Present results of all investigations of possible causes of heterogeneity among study results.	✓
	20d	Present results of all sensitivity analyses conducted to assess the robustness of the synthesized results.	✓
Reporting biases	21	Present assessments of risk of bias due to missing results (arising from reporting biases) for each synthesis assessed.	✓
Certainty of evidence	22	Present assessments of certainty (or confidence) in the body of evidence for each outcome assessed.	✓
**DISCUSSION**			13-17
Discussion	23a	Provide a general interpretation of the results in the context of other evidence.	✓
	23b	Discuss any limitations of the evidence included in the review.	✓
	23c	Discuss any limitations of the review processes used.	✓
	23d	Discuss implications of the results for practice, policy, and future research.	✓
**OTHER INFORMATION**			1, 6, 7
Registration and protocol	24a	Provide registration information for the review, including register name and registration number, or state that the review was not registered.	✓
	24b	Indicate where the review protocol can be accessed, or state that a protocol was not prepared.	✓
	24c	Describe and explain any amendments to information provided at registration or in the protocol.	✓
Support	25	Describe sources of financial or non-financial support for the review, and the role of the funders or sponsors in the review.	✓
Competing interests	26	Declare any competing interests of review authors.	✓
Availability of data, code and other materials	27	Report which of the following are publicly available and where they can be found: template data collection forms; data extracted from included studies; data used for all analyses; analytic code; any other materials used in the review.	✓

Supplementary Table 2: List of employed MeSH and Emtree terms

Search Concept Group	Keywords Provided	MeSH (PubMed/Medline)	Emtree (Embase)
**Prostate cancer / mCRPC**	metastatic castration-resistant prostate cancer / mCRPC / castration-resistant prostate cancer / advanced prostate cancer / metastatic prostate cancer	Prostatic Neoplasms [MeSH]; Neoplasm Metastasis [MeSH]; Drug Resistance, Neoplasm [MeSH]	prostatic neoplasm; metastatic tumor; castration resistance
**PSMA-targeted radioligand therapy**	prostate-specific membrane antigen radioligand therapy / PSMA radioligand therapy / PSMA-targeted radioligand therapy / PSMA-targeted radionuclide therapy / PSMA-targeted endoradiotherapy / PSMA-targeted therapy / PSMA-targeted alpha therapy / PSMA-targeted beta therapy	Prostate-Specific Membrane Antigen [MeSH]; Radionuclide Therapy [MeSH]; Receptors, Cell Surface [MeSH]	prostate specific membrane antigen; radionuclide therapy; radioligand therapy; cell surface receptor
**Emerging alpha-emitting PSMA**	[225Ac]Ac-PSMA-617 / 225Ac-PSMA-617 / actinium-225 PSMA / PSMA-targeted alpha therapy / PSMA-TAT / alpha-emitting PSMA radioligand	Actinium [MeSH]; Actinium Radioisotopes [MeSH]; alpha particles [MeSH]	actinium radioisotope; targeted alpha therapy
**Emerging beta / Auger PSMA (non-Lu)**	[161Tb]Tb-PSMA-617 / 161Tb-PSMA-617 / terbium-161 PSMA / terbium PSMA radioligand / [131I]PSMA / iodine-131 PSMA ligand	Terbium [MeSH]; Terbium Radioisotopes [MeSH]; Iodine Radioisotopes [MeSH]	terbium radioisotope; iodine-131 radioisotope; beta-emitting PSMA ligand; Auger-emitting PSMA ligand
**[177Lu]Lu / [225Ac]Ac-PSMA tandem therapy**	177Lu/225Ac-PSMA combination therapy / tandem PSMA radioligand therapy / dual-isotope PSMA therapy / 177Lu-PSMA plus 225Ac-PSMA	Lutetium [MeSH]; Actinium [MeSH]; Lutetium Radioisotopes [MeSH]; Actinium Radioisotopes [MeSH]; Radionuclide Therapy [MeSH]	lutetium radioisotope; actinium radioisotope; combination therapy; tandem radioligand therapy
**General PSMA ligands**	PSMA ligands / PSMA small-molecule ligands / PSMA-targeted small molecules / PSMA inhibitors	Molecular Probes [MeSH]; Ligands [MeSH]	small molecule; radiopharmaceutical; ligand
**Safety and efficacy outcomes**	adverse events / toxicity / treatment-related adverse events / hematologic toxicity / xerostomia / nephrotoxicity / PSA response / PSA50 / disease control rate / objective response / efficacy	Treatment Outcome [MeSH]; Drug-Related Side Effects and Adverse Reactions [MeSH]; Efficacy Analysis [MeSH]; Quality of Life [MeSH]	treatment outcome; adverse reaction

Supplementary Table 3: Rationale behind model selection approach implemented in this meta-analysis

Effect model	Utility	Reason for this approach	Supportive References
**Fixed effect**	In pooled meta-analysis of < 5 studies	With very few studies, the between-study variance is estimated very imprecisely, which can make random-effects results unstable. A fixed-effect model is more feasible as the primary analysis.	• DOI: 10.1002/9781119536604.ch10<br>• DOI: 10.1177/02537176251390987<br>• DOI: 10.48550/arXiv.2002.04211<br>• 10.4103/ija.ija_1203_24
**Random effects**	In pooled meta-analysis of ≥ 5 studies	With more studies, the between-study variance is more reliably estimable; a random-effects model is then justified because it acknowledges genuine variability in true effects across studies and yields appropriately wider, more conservative confidence intervals.	

Supplementary Table 4: Results of Methodological Quality evaluation by NIH evaluation tool. Q1, Was the research question or objective in this paper clearly stated?; Q2, Was the study population clearly specified and defined?; Q3, Was the participation rate of eligible persons at least 50%?; Q4, Were all the subjects selected or recruited from the same or similar populations (including the same time period) and were inclusion and exclusion criteria for being in the study prespecified and applied uniformly to all participants?; Q5, Was a sample size justification, power description, or variance and effect estimates provided?; Q6, For the analyses in this paper, were the exposure(s) of interest measured prior to the outcome(s) being measured?; Q7, Was the timeframe sufficient so that one could reasonably expect to see an association between exposure and outcome if it existed?; Q8, For exposures that can vary in amount or level, did the study examine different levels of the exposure as related to the outcome (e.g., categories of exposure, or exposure measured as continuous variable)? Q9, Were the exposure measures (independent variables) clearly defined, valid, reliable, and implemented consistently across all study participants?; Q10, Was the exposure(s) assessed more than once over time?; Q11, Were the outcome measures (dependent variables) clearly defined, valid, reliable, and implemented consistently across all study participants?; Q12, Were the outcome assessors blinded to the exposure status of participants?; Q13, Was loss to follow-up after baseline 20% or less?; Q14, Were key potential confounding variables measured and adjusted statistically for their impact on the relationship between exposure(s) and outcome(s)?

Item	1	2	3	4	5	6	7	8	9	10	11	12	13	14	Numerical Score	Quality score
Kratochwil 2017	Yes	No	Yes	Yes	Yes	No	Yes	No	Yes	Yes	Yes	NR	X	Yes	9	Good
Afshar-Oromieh 2017	Yes	Yes	X	Yes	No	Yes	Yes	Yes	Yes	No	Yes	No	Yes	No	9	Good
Kratochwil 2018	Yes	No	No	Yes	Yes	No	Yes	No	Yes	NA	Yes	NR	X	Yes	7	Fair
Sathekge 2019	Yes	Yes	NA	Yes	Yes	Yes	Yes	Yes	Yes	Yes	Yes	NR	Yes	Yes	12	Good
Khreish 2020	Yes	Yes	Yes	Yes	Yes	No	Yes	Yes	Yes	Yes	Yes	NR	Yes	Yes	12	Good
Sathekge 2020	Yes	No	NA	Yes	Yes	Yes	Yes	Yes	Yes	Yes	Yes	NR	Yes	Yes	11	Good
Yadav 2020	Yes	Yes	Yes	Yes	Yes	Yes	Yes	Yes	Yes	Yes	Yes	NR	X	Yes	12	Good
Satapathy 2020	Yes	Yes	Yes	Yes	Yes	No	No	No	Yes	No	Yes	NR	Yes	No	8	Fair
Sanli 2021	Yes	Yes	Yes	Yes	Yes	Yes	Yes	Yes	Yes	Yes	Yes	NR	Yes	Yes	13	Good
Rosar 2021a	Yes	No	NA	Yes	Yes	Yes	Yes	Yes	Yes	Yes	Yes	NR	No	Yes	10	Good
Rosar 2021b	Yes	No	NA	Yes	Yes	Yes	Yes	Yes	Yes	Yes	Yes	NR	NR	Yes	10	Good
Sen 2021	Yes	Yes	Yes	Yes	No	Yes	No	Yes	Yes	Yes	Yes	NR	Yes	Yes	13	Good
Zacherl 2021	Yes	Yes	NA	No	Yes	Yes	Yes	Yes	Yes	Yes	Yes	NR	Yes	Yes	10	Good
Van der Doelen 2021	Yes	No	Yes	NA	Yes	No	Yes	Yes	Yes	Yes	Yes	NR	Yes	Yes	10	Good
Sathekge 2022	Yes	No	No	Yes	No	Yes	No	Yes	Yes	Yes	Yes	NR	Yes	Yes	8	Fair
Ballal 2023	Yes	Yes	Yes	Yes	Yes	No	Yes	No	Yes	Yes	Yes	NR	Yes	Yes	11	Good
Laccetti 2023	Yes	Yes	X	Yes	Yes	Yes	Yes	Yes	Yes	Yes	Yes	No	Yes	X	11	Good
Schaeferâ€‘Schuler 2024	Yes	Yes	X	Yes	No	Yes	Yes	Yes	Yes	Yes	Yes	No	Yes	No	10	Good
Alan-Selcuk 2023	Yes	Yes	Yes	Yes	Yes	Yes	Yes	Yes	Yes	Yes	Yes	NR	Yes	Yes	13	Good
Sathekge 2023	Yes	No	NA	Yes	Yes	Yes	Yes	Yes	Yes	Yes	Yes	NR	Yes	Yes	11	Good
Feuerecker 2023	Yes	Yes	Yes	Yes	Yes	Yes	No	Yes	Yes	Yes	Yes	NR	No	Yes	11	Good
Sathekge 2024	Yes	Yes	Yes	Yes	Yes	Yes	Yes	Yes	Yes	Yes	Yes	Yes	Yes	Yes	13	Good
Rathke 2024	Yes	Yes	X	Yes	No	Yes	Yes	Yes	Yes	Yes	Yes	No	Yes	Yes	14	Good
Al-Ibraheem 2024	Yes	Yes	X	Yes	Yes	Yes	Yes	Yes	Yes	Yes	Yes	No	Yes	No	10	Good
Liu 2024	Yes	Yes	X	Yes	No	Yes	Yes	Yes	Yes	Yes	Yes	No	Yes	No	11	Good
Rosar 2024	Yes	Yes	X	Yes	No	Yes	Yes	Yes	Yes	Yes	Yes	No	Yes	Yes	11	Good
Rosar 2025	Yes	Yes	X	Yes	No	Yes	Yes	Yes	Yes	Yes	Yes	No	Yes	No	10	Good
Buteau 2025	Yes	Yes	X	Yes	Yes	Yes	Yes	Yes	Yes	Yes	Yes	No	Yes	Yes	12	Good
Kucuk 2025	Yes	Yes	X	Yes	No	Yes	Yes	Yes	Yes	Yes	Yes	No	Yes	No	12	Good
Ma 2025	Yes	Yes	X	Yes	No	Yes	Yes	Yes	Yes	Yes	Yes	No	Yes	Yes	10	Good
Widjaja 2025	Yes	Yes	X	Yes	No	Yes	Yes	Yes	Yes	Yes	Yes	No	No	No	10	Good
Sheikh 2025	Yes	Yes	X	Yes	No	Yes	Yes	Yes	Yes	Yes	Yes	No	Yes	Yes	10	Good

Supplementary Table 5: Characteristics of included studies. AU, Australia; CA, Canada; CH, Switzerland; CN, China; DE, Germany; IN, India; JO, Jordan; NL, Netherlands; OP, Original prospective; OR, Original retrospective; PSMA, Prostate-specific membrane antigen; TR, Turkey; TRT, Targeted radionuclide therapy; US, United States; ZA, South Africa.

Study	Country	Study Type	Center	TRT	Age	Sample Size	Median PSA	Cycles	Dosing regimen	Median Dose (KBQ/KG)	Prostatectomy (%)	ADT (%)	Prechemotherapy (%)	Radiotherapy(%)	Prior 177Lu-PSMA (%)	Prior 223Ra (%)	Heavy Pretreatment	Osseous Metastasis	Visceral Metastases	Nodal Metastases	ECOG >1
**Kratochwil 2017**	DE	OR	Unicentric	[^{225}Ac]-PSMA-617	68	14	118	39	Cyclic	100		93	93		23	15	1	1	9	11	
**Afshar-Oromieh 2017**	DE	OR	Unicentric	[131I]PSMA-1095	75	34	261	60	Cyclic	60							Unkown	28	3	18	
**Kratochwil 2018**	DE	OR	Unicentric	[^{225}Ac]-PSMA-617	70	40	169	120	Cyclic	100		85	75		0	25	1	97.5	16		8
**Sathekge 2019**	ZA	OR	Unicentric	[^{225}Ac]-PSMA-617	65	17	49.1	56	Cyclic	100	30	65	0	35	18		1	14	2	9	2
**Khreish 2019**	DE	OR	Unicentric	[^{177}Lu]/[^{225}Ac]-PSMA-617	72	20	215	20	Cyclic	50		100	65		100	4	1	19	6	9	8
**Sathekge 2020**	ZA	OR	Unicentric	[^{225}Ac]-PSMA-617	69	73	57.2	210	Cyclic	100	45	73	50	19	14	0	1	66	9		13

**Yadav 2020**	IN	OP	Unicentric	[^{225}Ac]-PSMA-617	70	28	222.2	85	Cyclic	100	11	54	93	43	54		1	27	6	24	
**Satapathy 2020**	IN	OR	Unicentric	[^{225}Ac]-PSMA-617	68	11	158	25	Cyclic	100		100	91	36	46		1	11	0	9	5
**Sanli 2021**	TR	OR	Unicentric	[^{225}Ac]-PSMA-617	70	12	129	25	Cyclic	100	25	100	75	50	58		1	12	2	8	6
**Rosar 2021**	DE	OR	Unicentric	[^{177}Lu]/[^{225}Ac]-PSMA-617	69	17	152	27	Cyclic	50	71	100	77	71	100	30	1	17	3	5	1
**Rosar 2021**	DE	OR	Unicentric	[^{177}Lu]/[^{225}Ac]-PSMA-617	77	15	272	20	Cyclic	50	47	100	67	53	0	20	1	15	9	11	3
**Sen 2021**	IN	OR	Unicentric	[^{225}Ac]-PSMA-617	68	38	147	48	Cyclic	100	24	64	100	16	24	5	1	34	7	17	
**Zacherl 2021**	DE	OR	Unicentric	[^{225}Ac]-PSMA-I&T	75	14	112	34	Cyclic	100	64	100	79	86	79	14	1	10	5	10	3
**Van der Doelen 2021**	NL	OR	Unicentric	[^{225}Ac]-PSMA-617	71	13	878	32	Cyclic	100		85	100		15	30	1	13	8	10	5
**Sathekge 2022**	ZA	OR	Unicentric	[^{225}Ac]-PSMA-617	63	53	466	167	Cyclic	100	59	100	0	21	0	0	1	47	7	36	11
**Laccetti 2023**	US	OP	Unicentric	[131I]PSMA-1095	70	9	28	13	Cyclic	60	55	89	33				0	2	0	2	
**Ballal 2023**	IN	OR	Unicentric	[^{225}Ac]-PSMA-617	67	56	337	204	Cyclic	100	21	89	100	94	51	0	1	51	18	43	17
**Alan-Selcuk 2023**	TR	OR	Unicentric	[^{225}Ac]-PSMA-617	70	23	103.8	38	Cyclic	100	48	100	96	70	100	0	1	21	8	13	
**Schaefer-Schuler 2023**	DE	OP	Unicentric	[161Tb]-PSMA-617	74	6	280	6	Cyclic	80	67	100	100	50	100		1	83		50	0
**Sathekge 2023**	ZA	OR	Unicentric	[^{225}Ac]-PSMA-617	67	21	196.7	68	Cyclic	100			0		0	0	Unkown	21	6	5	4
**Feuerecker 2023**	DE	OR	Unicentric	[^{225}Ac]-PSMA-617	69	21	202.3	21	Cyclic	100	67	100	100	67	100		1	21	8	9	1
**Sathekge 2024**	ZA	OR	Multicentric	[^{225}Ac]-PSMA-617	68	488	169.5	1174	Cyclic	100		80	66		32	4	1	435	107	352	170
**Rathke 2024**	CH	OR	Unicentric	[^{225}Ac]-PSMA-617	62	233	312	250	Fractionated	100	51	100	71	31	0		1	96	22	70	34
**Liu 2024**	CA	OP	Unicentric	[131I]PSMA-1095	74	11	162	22	Cyclic	60		78	100				0				2
**Al-Ibraheem 2024**	JO	OR	Unicentric	[161Tb]-PSMA-617	64	4	77.4	4	Cyclic	80	25	100	100	50	100		1	4	1	3	1
**Rosar 2024**	DE	OP	Unicentric	[^{177}Lu]/[^{225}Ac]-PSMA-617	75	33	416	45	Cyclic	50	33	100	64.5	42	0	9	1	32	24	12	14
**Perrone 2025**	DE	OR	Unicentric	[177Lu]/[225Ac]-PSMA-617	69	89		151	Cyclic	50	60.7	93.2	51.7	68.5	92.1		1	79	21	60	
**Sheikh 2025**	DE	OR	Unicentric	[177Lu]/[225Ac]-PSMA-617	75	25	132	65	Cyclic	50	60	80	92	68	72	20	1	25	5	22	
**Widjaja 2025**	DE	OR	Unicentric	[177Lu]/[225Ac]-PSMA-617	74	23	298	67	Cyclic	50	63	100	95		74		1	22	5	19	
**Ma 2025**	CN	OR	Unicentric	[225Ac]-PSMA-617	67	29	124.33	50	Cyclic	100	27.6	100	72.4		37.9		1	28	10	17	9
**Rosar 2025**	DE	OP	Unicentric	[161Tb]-PSMA-617	76	18	90	47	Cyclic	80	44	100	83	78	100	28	1	83	28	67	2
**Buteau 2025**	AU	OP	Unicentric	[161Tb]-PSMA-I&T	69	30	27	132	Cyclic	70	47	100	67	77			0	22	2	19	0
**Kucuk 2025**	TR	OP	Multicentric	[161Tb]-PSMA-617	71	7	42	14	Cyclic	80	57	100	100	57	100		1				1

Supplementary Table 6: Detailed Clinical and biochemical adverse events reported following PSMA RLT administration

Agent	Domain	Studies	Estimate (95% CI)	Heterogeneity		Egger's
				I^2	p-value	p-value
PSMA RLT	Overall	32	26% (22-31%)	96.3%	0.0001	0.57
	Xerostomia G1-2	28	68% (55-81%)	95.5%	0.0001	0.61
	Fatigue G1-2	8	55% (29-80%)	89.1%	0.0001	0.36
	Anemia G1-2	21	41% (27-55%)	94%	0.0001	0.52
	GIT G1-2	8	31% (19-45%)	75.5%	0.0001	0.29
	Leukopenia G1-2	20	21% (14-30%)	86%	0.0001	0.54
	Thrombocytopenia G1-2	18	21% (12-30%)	88.4%	0.0001	0.41
	Nephrotoxicity G1-2	12	20% (8-35%)	93.3%	0.0001	0.57
	Hepatotoxicity G1-2	4	19% (6-38%)	N/A	N/A	0.49
	Xerostomia G3	7	11% (4-22%)	79.9%	0.0001	0.28
	Anemia G3-4	18	10% (7-14%)	49.6%	0.06	0.33
	Bone pain flare	4	10% (2-21%)	N/A	N/A	0.12
	Thrombocytopenia G3-4	14	7% (4-10%)	37.9%	0.09	0.74
	Nephrotoxicity G3-4	9	4% (2-7%)	23.3%	0.24	0.82
	Fatigue G3-4	2	3% (1-10%)	N/A	N/A	N/A
	Leukopenia G3-4	10	3% (2-6%)	28.4%	0.18	0.25
$[^{225}Ac]$Ac-PSMA	Overall	18	28% (21-34%)	97.2%	0.0001	0.18
	Xerostomia G1-2	18	82% (70-92%)	92.5%	0.0001	0.45
	Fatigue G1-2	3	68% (49-84%)	N/A	N/A	0.89
	GIT G1-2	4	44% (32-56%)	N/A	N/A	0.76
	Anemia G1-2	13	40% (21-60%)	95.7%	0.0001	0.18
	Nephrotoxicity G1-2	5	24% (7-48%)	96.2%	0.0001	0.73
	Leukopenia G1-2	12	18% (9-29%)	88.5%	0.0001	0.32
	Thrombocytopenia G1-2	11	17% (6-32%)	92.4%	0.0001	0.69
	Hepatotoxicity G1-2	3	13% (6-22%)	N/A	N/A	0.17
	Xerostomia G3	6	12% (3-24%)	85.3%	0.0001	0.44
	Anemia G3-4	10	9% (4-15%)	63.7%	0.0001	0.35
	Thrombocytopenia G3-4	7	5% (2-9%)	29.2%	0.21	0.98
	Nephrotoxicity G3-4	7	3% (2-5%)	11%	0.87	0.52
$[^{177}Lu]$Lu/$[^{225}Ac]$Ac-PSMA	Overall	7	20% (10-33%)	96.2%	0.0001	0.44
	Xerostomia G1-2	7	42% (5-84%)	98.2%	0.0001	0.23
	Anemia G1-2	3	35% (8-68%)	N/A	N/A	0.81
	Thrombocytopenia G1-2	2	23% (15-31%)	N/A	N/A	N/A
	Leukopenia G1-2	2	22% (14-30%)	N/A	N/A	N/A
	Nephrotoxicity G1-2	3	11% (5-19%)	N/A	N/A	0.63
	Anemia G3-4	5	10% (5-17%)	26.7%	0.24	0.48
	Thrombocytopenia G3-4	3	6% (2-11%)	N/A	N/A	0.29
	Leukopenia G3-4	2	3% (1-7%)	N/A	N/A	N/A
$[^{161}Tb]$Tb-PSMA	Overall	5	24% (14-33%)	23.8%	0.38	0.89
	Anemia G1-2	3	56% (27-82%)	N/A	N/A	0.46
	Fatigue G1-2	3	55% (10-99%)	N/A	N/A	0.83
	Xerostomia G1-2	2	54% (33-75%)	N/A	N/A	N/A
	Leukopenia G1-2	3	31% (18-45%)	N/A	N/A	0.18
	Thrombocytopenia G1-2	2	25% (13-38%)	N/A	N/A	N/A
	Nephrotoxicity G1-2	4	25% (1-69%)	N/A	N/A	0.87
	Anemia G3-4	2	16% (3-35%)	N/A	N/A	N/A
	Thrombocytopenia G3-4	2	11% (1-29%)	N/A	N/A	N/A
	Bone pain flare	3	10% (1-23%)	**N/A**	N/A	0.16
	GIT G1-2	2	10% (3-21%)	N/A	N/A	N/A
$[^{131}I]$PSMA	Overall	3	25% (14-38%)	N/A	N/A	0.93
	Xerostomia G1-2	3	51% (6-95%)	N/A	N/A	0.37
	Leukopenia G1-2	3	32% (1-84%)	N/A	N/A	0.12
	Thrombocytopenia G1-2	3	29% (17-43%)	N/A	N/A	0.38
	GIT G1-2	2	24% (7-46%)	N/A	N/A	N/A
	Thrombocytopenia G3-4	2	19% (8-32%)	N/A	N/A	N/A
	Fatigue G1-2	2	13% (4-26%)	N/A	N/A	N/A
	Leukopenia G3-4	2	6 (0-16%)	N/A	N/A	N/A
